# Local and long-range GABAergic circuits in hippocampal area CA1 and their link to Alzheimer’s disease

**DOI:** 10.3389/fncir.2023.1223891

**Published:** 2023-09-29

**Authors:** Melissa Hernández-Frausto, Olesia M. Bilash, Arjun V. Masurkar, Jayeeta Basu

**Affiliations:** ^1^Neuroscience Institute, New York University Langone Health, New York, NY, United States; ^2^Department of Neuroscience and Physiology, New York University Grossman School of Medicine, New York, NY, United States; ^3^Meinig School of Biomedical Engineering, Cornell University, Ithaca, NY, United States; ^4^Center for Cognitive Neurology, Department of Neurology, New York University Grossman School of Medicine, New York, NY, United States; ^5^Department of Psychiatry, New York University Grossman School of Medicine, New York, NY, United States; ^6^Center for Neural Science, New York University, New York, NY, United States

**Keywords:** GABAergic circuits, Alzheimer’s disease, hippocampus (CA1), long-range GABAergic neurons, disinhibition

## Abstract

GABAergic inhibitory neurons are the principal source of inhibition in the brain. Traditionally, their role in maintaining the balance of excitation-inhibition has been emphasized. Beyond homeostatic functions, recent circuit mapping and functional manipulation studies have revealed a wide range of specific roles that GABAergic circuits play in dynamically tilting excitation-inhibition coupling across spatio-temporal scales. These span from gating of compartment- and input-specific signaling, gain modulation, shaping input–output functions and synaptic plasticity, to generating signal-to-noise contrast, defining temporal windows for integration and rate codes, as well as organizing neural assemblies, and coordinating inter-regional synchrony. GABAergic circuits are thus instrumental in controlling single-neuron computations and behaviorally-linked network activity. The activity dependent modulation of sensory and mnemonic information processing by GABAergic circuits is pivotal for the formation and maintenance of episodic memories in the hippocampus. Here, we present an overview of the local and long-range GABAergic circuits that modulate the dynamics of excitation-inhibition and disinhibition in the main output area of the hippocampus CA1, which is crucial for episodic memory. Specifically, we link recent findings pertaining to GABAergic neuron molecular markers, electrophysiological properties, and synaptic wiring with their function at the circuit level. Lastly, given that area CA1 is particularly impaired during early stages of Alzheimer’s disease, we emphasize how these GABAergic circuits may contribute to and be involved in the pathophysiology.

## Introduction

Gamma-aminobutyric acid (GABA) is the primary inhibitory neurotransmitter in the mammalian central nervous system. It is released by GABAergic inhibitory neurons (INs), which serve as one of the main sources of inhibition (Caputi et al., 2013; Le Magueresse and Monyer, 2013). GABAergic INs modulate the activity of other neurons to maintain a homeostatic excitation-inhibition balance. This is a striking feature of the cortex where E/I ratios are tightly maintained within various layers (Pfeffer et al., 2013; Xue et al., 2014; Adesnik, 2018; Yen et al., 2022). Do GABAergic inputs just act to balance excitation, or do they have specific roles in organizing information flow? The balance of excitation and inhibition is critical for normal brain function. Disruptions to excitation-inhibition balance can result in hyperexcitability, runaway excitation, and disturbance of oscillatory synchrony, which can be seen in epilepsy, neuropsychiatric disorders such as post-traumatic stress disorder (PTSD), schizophrenia, anxiety, and depression (Marín, 2012), and neurodegenerative diseases such as Alzheimer’s disease (AD; Han et al., 2012; Busche et al., 2015; Busche and Konnerth, 2016; O’Donnell et al., 2017; Bridi et al., 2020). However, during learning, shifts in the weight of excitation and inhibition are important to discriminate and store only the relevant information based on contexts. A precise model of how excitatory and inhibitory neurons cooperate to tune information flow is crucial to our understanding of the brain. In this context, hippocampal area CA1 is particularly interesting to study- it provides the main output of the hippocampus and thus critical for memory-guided behavior. Interestingly, CA1 is also the part of hippocampus affected earliest and the most in AD (Braak and Braak, 1991).

A single hippocampal CA1 pyramidal neuron receives several different inputs (glutamatergic, GABAergic) from various sources (hippocampus, entorhinal cortex, prefrontal cortex). Glutamatergic inputs drive excitation in a circuit; GABAergic inputs typically inhibit the propagation of excitation. ***How do local and long-range GABAergic circuits interact to change the dynamics of excitation and inhibition for acquiring important information?*** The hippocampal CA1 region alone has >21 different types of local GABAergic neurons with distinct molecular make-up and physiological properties. However, we know little about how these GABAergic neurons contribute to hippocampal functions such as plasticity or learning behavior.

Several lines of evidence suggest that local and long-range circuit interactions between pyramidal neurons (PNs) and GABAergic INs are poised to play a prominent role in higher-order cognitive functions. Within the hippocampus, dynamically controlling the excitation-inhibition (E/I) balance – tilting it in favor of excitation or inhibition in a context-specific manner at the single cell and network levels – can influence memory processing, multisensory coding, and fine-tuning of behaviorally-relevant neuronal activity (Klausberger and Somogyi, 2008; Joshi et al., 2017; Szabo et al., 2022). For example, functional interactions between the prefrontal cortex, entorhinal cortex, and hippocampus that support formation of episodic memories of context and events in the hippocampus rely on the activity of long-range GABAergic projection neurons from the cortex and local GABAergic microcircuits in the hippocampus (Basu et al., 2013, 2016; Malik et al., 2022).

At the single-neuron level, the distribution of specific types of inhibitory synapses varies along the somato-dendritic axis of pyramidal neurons found in hippocampal area CA1, even within a dendritic branch (Megías et al., 2001; Bloss et al., 2016; Cembrowski et al., 2016). Surprisingly, the electrophysiological, neurochemical, and functional characteristics of the inhibitory synapses correlate with their axo-dendritic distribution. At the network level, the interaction between local and long-range excitation and inhibition in the cortex and hippocampus could be important for supporting context-dependent stability and flexibility of memory representations encoding familiar and novel experiences and generating adaptive learned behaviors. Furthermore, GABAergic circuits may substantially coordinate oscillations (Somogyi et al., 2014), such as gamma oscillations during learning (Wulff et al., 2009), theta oscillations during locomotion (Csicsvari et al., 1999; Buzsáki, 2002; Bezaire et al., 2016; Melzer and Monyer, 2020), and sharp wave ripples (SWR; Cutsuridis and Taxidis, 2013; Schlingloff et al., 2014; Stark et al., 2014; Evangelista et al., 2020; Noguchi et al., 2022) in quiet wakefulness, and sleep (Eichenbaum, 2000; Squire, 2004; Basu and Siegelbaum, 2015; Francavilla et al., 2018; Eyre and Bartos, 2019; Udakis et al., 2020; Malik et al., 2022; Yen et al., 2022).

In the present review, we describe the local and long-range GABAergic circuits in hippocampal area CA1. However, rather than describing inhibitory neuron types based on their expressed molecular markers, we focus on a functional classification approach. We present our perspective on how specific inhibitory microcircuits modulate compartment-specific activity, as well as how the dynamic interaction between excitation, inhibition, and disinhibition shapes dendritic integration, plasticity, and behavior. We particularly showcase the recently described GABAergic disinhibitory circuit motifs to emphasize the role of ‘inhibitory’ neurons in boosting excitatory signaling rather than curbing it, and coordinating long-range inter-regional interactions beyond local ‘interneuron’ domains. Finally, we highlight how these circuits may contribute to the neurodegeneration seen during Alzheimer’s disease.

## General GABAergic circuit motifs in CA1

Within the hippocampus, local inhibition is mediated by GABAergic microcircuits comprising of INs that target PNs directly to suppress their activity. Within these GABAergic microcircuits that drive inhibition there are two organizational motifs.

### Feed-forward inhibition

**Figure 1:**
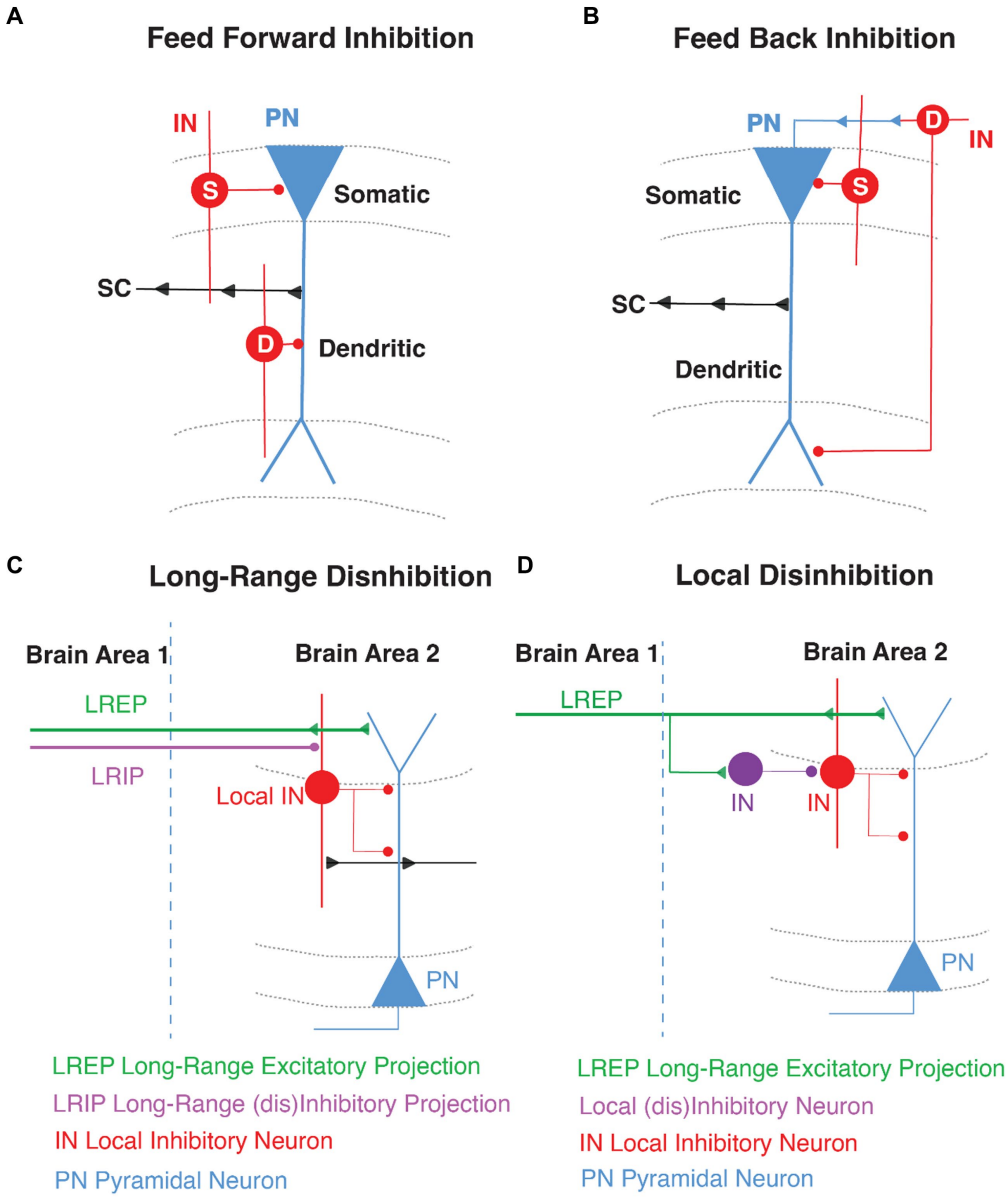
General GABAergic Circuit Motifs in area CA1. Schematic representations of **(A)** feed-forward inhibition (FFI) and **(B)** feed-back inhibition (FBI) in CA1 highlighting the pyramidal neuron (PN, in blue) with the Schaffer Collateral inputs (SC, arrows in black) and the inhibition of local interneurons (IN, in red) in somatic (S) and dendritic (D) compartments. **(C)** Long-range disinhibition **(D)** Local disinhibition in hippocampal area CA1.

Within hippocampal area CA1, feed-forward inhibition (FFI) is locally mediated by INs that are directly excited by the glutamatergic inputs arriving from the EC or by intra-hippocampal glutamatergic inputs from CA3 or CA2 (Chevaleyre and Siegelbaum, 2010; Basu and Siegelbaum, 2015; Ferrante and Ascoli, 2015; Zemla and Basu, 2017; Bilash et al., 2023). These INs then target PNs to limit their activity and the propagation of information (Buzsaki, 1984; Pouille and Scanziani, 2001; Price et al., 2008; Kullmann, 2011; Basu et al., 2013, 2016; Figure 1).

### Feed-back inhibition

Feedback inhibition (FBI) occurs when CA1 PNs recurrently target INs through direct monosynaptic connections and the INs in turn target the PNs (Tremblay et al., 2016). Thus, FBI is driven by recurrent excitation and the overall effect is auto-inhibition of PN activity. The initial drive for the PN spiking to trigger FBI arises from strong activity of the proximal CA3 or CA2 excitatory inputs and on occasion from distal EC (Buzsaki, 1984; Kullmann, 2011; Tyan et al., 2014; Figure 1).

### Disinhibition

Disinhibition is a circuit motif that results from an IN directly targeting another IN, which releases the inhibition from a downstream PN (Basu et al., 2013, 2016; Francavilla et al., 2015; Guet-McCreight et al., 2020; Bilash et al., 2023). So rather than inhibiting a PN, the GABAergic IN-specific-IN (ISI) mediates inhibition of inhibition and thereby disinhibition of coincident excitatory transmission. In the hippocampus, disinhibitory gating can be local (LEC driven VIP INs) or long-range (direct GABAergic projections from EC). Curiously, most long-range GABAergic projection neurons synapse onto INs in their target region and are therefore primarily disinhibitory in nature (Fuchs et al., 2007; Melzer et al., 2012; Caputi et al., 2013; Tyan et al., 2014; Basu et al., 2016; Melzer and Monyer, 2020; Yen et al., 2022; Figure 1).

### Hippocampal circuit organization and layer specific inhibition

**Figure 2:**
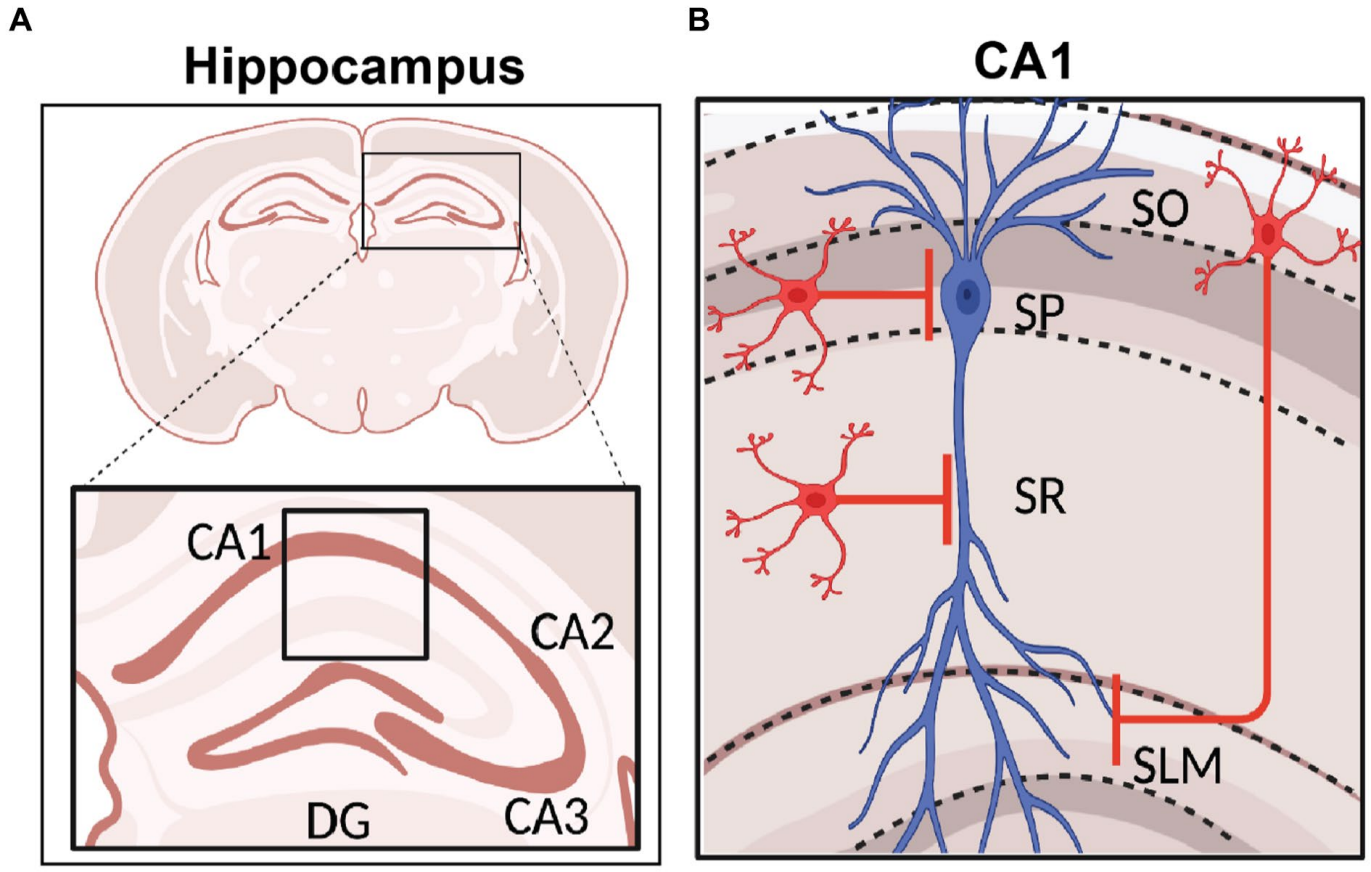
Hippocampal Formation and area CA1 Circuitry. **(A)** Schematic representation of coronal section of a rodent brain highlighting the hippocampal region with its sub-areas dentate gyrus (DG), CA3, CA2, and CA1. **(B)** Stratification of hippocampal area CA1 namely stratum oriens (SO), *stratum pyramidale* (SP), *stratum radiatum* (SR), and *stratum lacunosum moleculare* (SLM). The CA1 pyramidal neuron soma and dendrites organization are shown in blue, and INs in red. Created with BioRender.

The hippocampus is anatomically divided into different sub-regions, namely dentate gyrus (DG), CA3, CA2, CA1 and subiculum, which are each believed to perform distinct roles in memory operations. Each of these subregions have a laminar organization through the dorso-ventral axis (Amaral and Witter, 1989; Zemla and Basu, 2017). Hippocampal area CA1 consists of four layers. The pyramidal cell layer, or *stratum pyramidale*, is where all the PN somata are located. These PNs are innervated exclusively by GABAergic synapses from perisomatic basket interneurons. The *stratum oriens* (SO) is where the basal dendrites and axonal arborizations of CA1 PNs are located. SO hosts oriens lacunosom moleculare (OLM) interneurons and basket cell soma and axo-axonic interneurons (Maccaferri, 2005), and receives input from area CA2 (Chevaleyre and Siegelbaum, 2010), and medial septum (Lovett-Barron et al., 2014). The *stratum radiatum* (SR), where PN apical dendrites are located, receives excitatory input from CA3 PNs via the Schaffer Collateral (SC) synapses and is embedded with some bistratified dendrite-targeting GABAergic neurons. Lastly, the *stratum lacunosum moleculare* (SLM), where most distal tuft dendrites are located, receives input from the entorhinal cortex (EC) and has cell bodies of several dendrite-targeting and some soma-targeting INs (Amaral and Witter, 1989; Witter et al., 2000; Basu and Siegelbaum, 2015). The SR/SLM border region is particularly rich in GABAergic neuron cell bodies that control the flow of direct cortical inputs arriving distally upon CA1 PNs and their integration with proximal inputs carrying content processed through the indirect intra-hippocampal trisynaptic circuit (DG → CA3 → CA1). Both the direct EC and indirect trisynaptic pathways have been implicated in learning and memory storage (Barnes et al., 1977; Van Strien et al., 2009; Basu et al., 2013, 2016; Jonas and Lisman, 2014; Eyre and Bartos, 2019; Figure 2).

## Classification of GABAergic interneurons

GABAergic INs constitute ~10-20% of all neurons in cortex (Bartos et al., 2007; Klausberger and Somogyi, 2008; Caputi et al., 2013) and ~10–15% of all neurons in the hippocampus (Bezaire and Soltesz, 2013; Pelkey et al., 2017). Within the hippocampus, area CA1 contains more than 21 types of GABAergic INs (Klausberger and Somogyi, 2008). Each of these GABAergic IN types can be classified based on single cell transcriptome analyses and according to their morphology, molecular markers, intrinsic physiological properties, postsynaptic target cells, developmental origin, and function in the adult brain (Ascoli et al., 2008; Klausberger and Somogyi, 2008; Le Magueresse and Monyer, 2013; Pelkey et al., 2017; Harris et al., 2018). However, the integrated classification of interneuron types remains a daunting challenge in cellular and circuit neuroscience (Ascoli et al., 2008; Kepecs and Fishell, 2014; Pelkey et al., 2017; Zeng, 2022). Circuit mapping studies have greatly benefitted from classifying INs based on the expression of specific molecular markers, and the development of transgenic animals based on these molecular markers has provided a relatively precise and consistent strategy to label and manipulate major interneuron populations selectively (Taniguchi et al., 2011; Dimidschstein et al., 2016).

**Figure 3:**
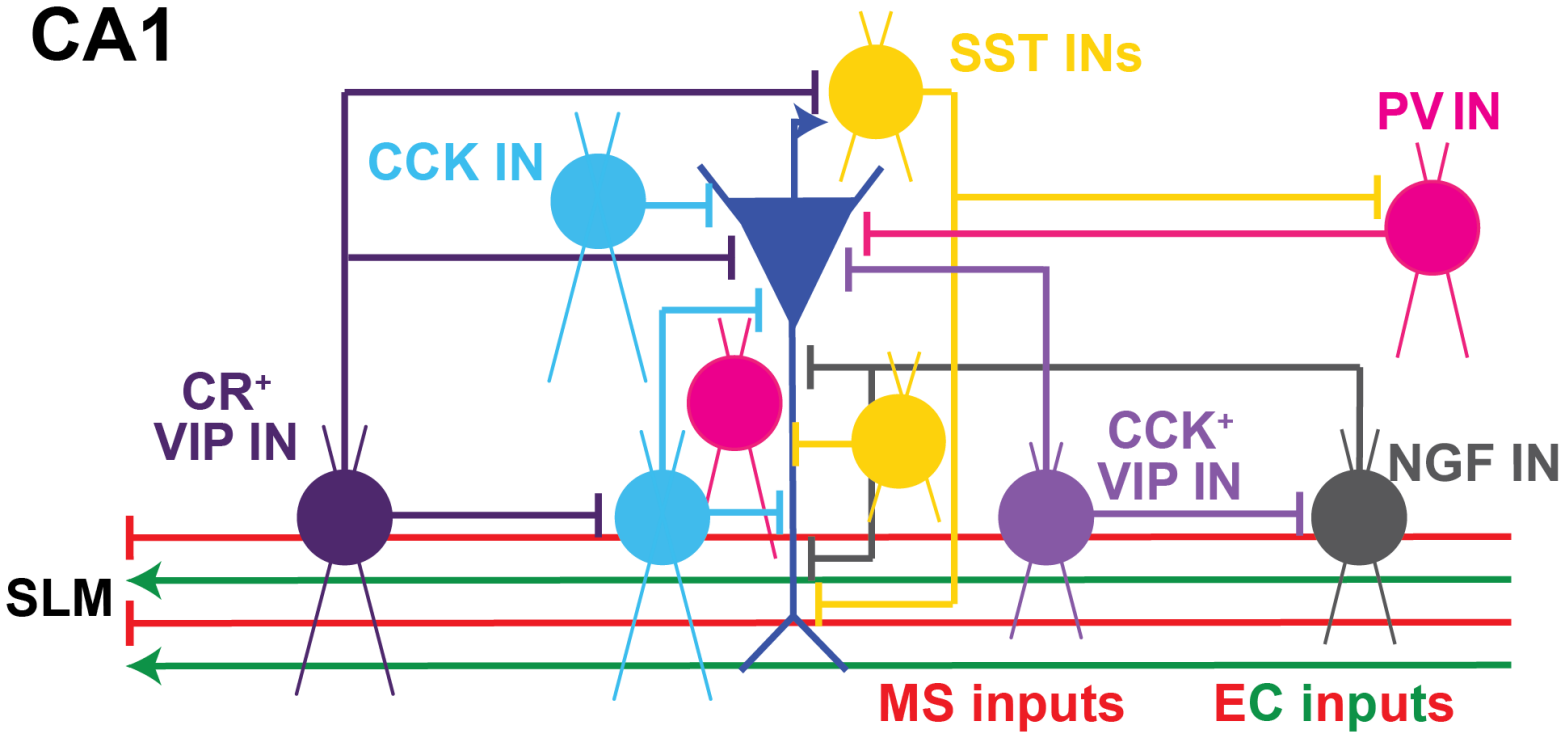
Local Inhibitory microcircuits in area CA1. Schematic representation of CA1 microcircuit connectivity, the CA1 pyramidal neuron (blue) is surrounded by local GABAergic microcircuitry: PV^+^ IN (pink), SST^+^ IN (yellow), soma-targeting CCK^+^ basket cell and SR/SLM border CCK IN (light blue), CR^+^ VIP IN (dark purple), CCK^+^ VIP^+^ IN (light purple), and NPY^+^ IN (gray). Excitatory and inhibitory inputs from EC into SLM of area CA1 shown in green and red, respectively. Inhibitory inputs from the Medial septum (MS) into SLM of area CA1 shown in red.

In terms of their expression of molecular markers, GABAergic INs have been traditionally categorized as parvalbumin (PV), vasoactive intestinal peptide (VIP), cholecystokinin (CCK), somatostatin (SST), neuropeptide Y (NPY), and neuron-derived neurotrophic factor (NDNF) and/or nitric oxide synthase (nNOS), among others. These interneuron types generally possess characteristic intrinsic properties and short-term synaptic plasticity dynamics due to their unique molecular make up. Moreover, they can perform specific functions in modulating compartment-specific and behaviorally-relevant activity based on their layer specificity location, connectivity onto downstream PNs and responses to neuromodulators (Figure 3). Each of these interneuron populations can be further subdivided based on co-expressed molecular markers, intrinsic properties, morphology, location within a particular CA1 layer, or input–output connectivity [thoroughly reviewed in (Pelkey et al., 2017)]. Here we provide a brief overview of these sub-classes.

### PV INs

Parvalbumin-expressing (PV) INs are classically considered to provide perisomatic inhibition onto a neighboring CA1 PN by surrounding its soma with a basket-like axon morphology (Sik et al., 1995; Cope et al., 2002; Pawelzik et al., 2002; Klausberger et al., 2005; Takács et al., 2015; Dudok et al., 2021a). Moreover, PV INs can also have bistratified morphological subtypes that specifically target the proximal apical dendrites to mediate dendritic feed-forward inhibition (FFI; Basu et al., 2013, 2016; Udakis et al., 2020; Bilash et al., 2023; Chamberland et al., 2023). PV INs also participate in FBI circuit motifs. Few recurrent collaterals of CA1 PNs make functional synaptic contacts onto PV basket cells that target basal dendrites in *stratum oriens* (Ribak et al., 1993; Hu et al., 2014). Furthermore, the apical dendrite targeting OLM neurons, which are a classical FBI also express low levels of parvalbumin (Klausberger, 2009). Recent studies in CA1 show that PV INs particularly target PNs in the deep layer, while being driven by PNs the superficial layer (Lee A. T. et al., 2014). Lastly, parvalbumin is also expressed in axo-axonic interneurons that directly inhibit the axon initial segment of a pyramidal neuron (Pawelzik et al., 2002; Takács et al., 2015). Functionally, fast-spiking PV INs in CA1 are involved in networking oscillations modulating the PN synchrony particularly in the gamma frequency regime (English et al., 2017) and spatial working memory (Murray et al., 2011; Hu et al., 2014; Harris et al., 2018). PV IN synapses undergo inhibitory long-term depression (i-LTD) during theta burst stimulation (TBS) as well as a spike-timing-dependent plasticity (STDP) paradigm, which is mediated via the activation of GABA_A_ receptors and T-type voltage-gated calcium channels (VGCCs; Udakis et al., 2020). In area CA1, the activity of PV INs is modulated by opioids, specifically via the μ-opioid receptors (Glickfeld et al., 2008). Furthermore, neuromodulation of PV INs is mediated by oxytocin, which increases PV IN firing rate through GABA_A_ receptors (Owen et al., 2013) and by D4-dopamine receptors that enhances the CA3 SC input-driven from CA1 to mediate PV FFI upon PNs to suppress SC pathway output (Rosen et al., 2015).

### CCK INs

Cholecystokinin-expressing (CCK) INs were classically considered to mediate FBI (Glickfeld and Scanziani, 2006) at the CA1 pyramidal neuron soma because of their slower responses compared to PV basket cells. Furthermore, these INs are known for their asynchronous GABA release (Hefft and Jonas, 2005), where they fail to keep up with fidelity during high-frequency trains of activation. However, recent studies using optogenetic activation and pharmacogenetic silencing (Basu et al., 2013) show that CCK INs are recruited in a FFI manner by the Schaffer Collateral inputs from CA3, as well as by the perforant path inputs from the entorhinal cortex. In fact, CCK INs can mediated robust and fast FFI both at the soma and dendrites to considerably suppress the amplitude of coincident EPSPs. *In vivo* juxtacellular recordings of single CCK INs in area CA1 reveals their preferred theta phase firing precedes that of CA1 PNs, putting them in a strategic position to modulate place cell firing at the peak of the theta cycle (Klausberger and Somogyi, 2008). Recent *in vivo* two-photon imaging studies show that CCK INs (Geiller et al., 2020; Dudok et al., 2022; Dudok and Soltesz, 2022) are particularly active during rest or when animals are stationary, creating a potential functional link to quiet, awake behavioral states associated with sharp wave ripples (Buzsáki, 2015). A well-characterized feature of the CCK INs, is their sensitivity to cannabinoid modulation by virtue of the cannabinoid 1 receptors (CB1Rs) they express (Chevaleyre and Castillo, 2003; Glickfeld and Scanziani, 2006; Freund and Katona, 2007; Castillo et al., 2012). Release of retrograde endocannabinoid messengers upon activation and depolarization of CA1 PNs allows for local and rapid suppression of GABA release from CCK basket cells, which are particularly enriched in CB1Rs (Freund and Katona, 2007). This can occur across shorter time scales, e.g., depolarization induced suppression of inhibition (DSI; Wilson et al., 2001; Yoshida et al., 2002) as well as longer time scales, e.g., inhibitory long-term depression (iLTD) time scales (Chevaleyre et al., 2007; Heifets and Castillo, 2009; Castillo et al., 2012; Basu et al., 2013). Notably, the genetic targeting of CCK INs has been challenging considering CCK pre- pro- hormone is widely expressed in glutamatergic pyramidal neurons as well. Thus, targeting CCK INs exclusively requires intersectional strategies (Taniguchi et al., 2011; Basu et al., 2013, 2016). Finally, recent studies suggest that immediate early gene NPAS4 preferentially modulates CCK IN activity and synaptic output during exposure to enriched environments (Hartzell et al., 2018) and contextual fear learning (Sun et al., 2020).

### SST INs

Somatostatin-expressing (SST) INs classically mediate dendritic inhibition onto pyramidal neurons either in a FFI or FBI manner (Oliva et al., 2000; Leão et al., 2012; Lovett-Barron et al., 2012, 2014; Müller and Remy, 2014; Udakis et al., 2020). SST INs in area CA1 consist of two main subpopulations: OLM INs, which mediate FBI (Leão et al., 2012), and bistratified SST INs, which modulate CA3-driven FFI (Lovett-Barron et al., 2012). OLM INs are recruited by spiking CA1 PNs and inhibit the distal dendrites of CA1 PNs. OLM neurons modulate SC input plasticity (Leão et al., 2012) and gate EC inputs that are relevant for contextual fear learning (Cutsuridis et al., 2010; Lovett-Barron et al., 2014). The OLM neurons have been more extensively studied, and can be characterized by the specific expression of nicotinic acetylcholine receptor α2 subunit (CHRNA2; Leão et al., 2012; Lovett-Barron et al., 2014). Their cell bodies are located in the oriens layer with the axons projecting to the *stratum lacunosum moleculare*, and known to be modulated by cholinergic input (Leão et al., 2012; Lovett-Barron et al., 2014), and the effect of this modulation is amplified by gap junctions interconnecting the OLM cells. SST IN synapses upon CA1 PN have also been shown to undergo LTP during theta burst stimulation (Udakis et al., 2020) through voltage-gated T- and L-type calcium channel modulation. Imaging studies comparing SST IN and PV IN activity in animals navigating between familiar and novel environments, have shown that the activity of SST INs is transiently suppressed when animals are exposed to novel environments to disinhibit dendrites, while somatic inhibition is dialed up by boosting PV IN activity (Sheffield et al., 2017).

### VIP INs

Vasoactive intestinal peptide-expressing (VIP) INs generally target other inhibitory neurons, thereby disinhibiting downstream pyramidal neurons (Acsády et al., 1996; Chamberland and Topolnik, 2012). They have an important role in the modulation of distal dendritic inhibition of PNs with specific target of bistratified and oriens INs controlling the firing rate and FFI (Tyan et al., 2014). They are strongly stimulated by cholinergic modulation (Bell et al., 2015; Askew et al., 2019; Ren et al., 2022) through α4β2 nicotinic acetylcholine (Ach) receptors (Bell et al., 2015). VIP INs can be divided into sub-populations based on their additional expression of calretinin (CR), CCK, or muscarinic acetylcholine receptor 2 (Tyan et al., 2014; Francavilla et al., 2018; Guet-McCreight et al., 2020). Genetically targeting these VIP IN subtypes requires intersectional tools, so dissociating their specific functions has been challenging. Nevertheless, a couple of recent studies have used intersectional tools and modeling to dissociate the function of VIP/CR+ and VIP/CCK INs. The VIP/CR+ INs are primarily disinhibitory and target a host of different interneurons types in area CA1, particularly the dendrite-targeting OLM INs (Tyan et al., 2014; Bilash et al., 2023). On the other hand, the CCK-expressing VIP INs can target PNs and mediate perisomatic FFI (Turi et al., 2019; Guet-McCreight et al., 2020; Kullander and Topolnik, 2021) through asynchronous GABA release (Tyan et al., 2014). For detailed information regarding their disinhibitory function please see Disinhibitory GABAergic Circuits section.

### Neurogliaform cells – INs expressing NDNF, NPY, and/or nNos

Neurogliaform cells (NGF) express neuron-derived neurotrophic factor (NDNF) but can also be defined by NPY and/or nNOS expression (Price et al., 2005; Tricoire et al., 2010; Armstrong et al., 2012; Milstein et al., 2015; Tasic et al., 2016; Guo et al., 2021). NGF INs soma are predominantly restricted to the distal dendritic SLM layer in CA1, with a smaller distinct subset residing at the border of SLM/SR (Capogna, 2011). Given their position, NGF INs are likely predominantly driven by glutamatergic inputs from EC, as well as thalamus. However, NPY+ NGF neurons have been shown to modulate integration of SC and EC inputs (Milstein et al., 2015). A unique feature of NGF INs is their volume transmission of GABA to provide slow-acting inhibition, via activation of both GABA_A_ and GABA_B_ receptors onto neurons within a particular radius (Price et al., 2005, 2008; Tasic et al., 2016; Abs et al., 2018; Mercier et al., 2022).

In conclusion, molecular markers can be a helpful tool to classify the different GABAergic INs and determine their modulation of excitation-inhibition balance. Nevertheless, they can co-express similar molecular markers and have specific locations throughout the various layers of area CA1. For instance, the expression of molecular markers PV, SST, or NPY can overlap in morphologically-defined *oriens-lacunosum moleculare* (OLM) interneurons or bistratified neurons (Klausberger and Somogyi, 2008; Klausberger, 2009; Katona et al., 2014; Müller and Remy, 2014). This calls for IN classification and nomenclature systems to use a combination of the molecular markers and their localization in laminar stratification of hippocampal area CA1 (Figure 3).

## GABAergic microcircuits in modulating compartment-specific activity in hippocampal area CA1

Synaptic inhibition can powerfully influence the dendritic and somatic activity of a pyramidal neuron (PN; Miles et al., 1996; Gidon and Segev, 2012; Müller et al., 2012; Pouille et al., 2013; Marlin and Carter, 2014; Müllner et al., 2015). Arranged in specialized microcircuit motifs, interneurons can provide axonal (Kiss et al., 1996; Dudok et al., 2021b), perisomatic (Pawelzik et al., 2002; Klausberger et al., 2005; Dudok et al., 2021a), or dendritic (Lacaille and Schwartzkroin, 1988; Klausberger, 2009; Lovett-Barron et al., 2012; Müller and Remy, 2014) inhibition onto specific compartments of CA1 pyramidal neurons. Other interneurons specifically target GABAergic interneurons, thereby serving a disinhibitory function within the larger neural circuit in area CA1 (Acsády et al., 1996; Chamberland and Topolnik, 2012). GABAergic synapses that densely surround the perisomatic region of a CA1 PNs strongly modulate somatic output (Pouille and Scanziani, 2001), while those found along the dendritic tree can serve to precisely modulate EC- or CA3-driven dendritic computations (Klausberger, 2009; Lovett-Barron et al., 2012; Basu et al., 2013, 2016; Bloss et al., 2016; Schulz et al., 2018; Bilash et al., 2023; Chamberland et al., 2023). Ultimately, by affecting the entire span of a CA1 pyramidal neuron (Buhl et al., 1994; Glickfeld et al., 2009; Bloss et al., 2016), GABAergic INs can serve as powerful circuit switches to rapidly, precisely, and flexibly shape single-cell and network computations (Lovett-Barron et al., 2012; Royer et al., 2012; Milstein et al., 2015; Basu et al., 2016; Grienberger et al., 2017).

PV INs mediate FFI in area CA1, modulating somatic and axonal activity in an apparent temporarily silent state (Sik et al., 1995; Pawelzik et al., 2002; Takács et al., 2015; Pelkey et al., 2017; Dudok et al., 2021a; Chamberland et al., 2023). On the other hand, CCK IN subpopulations provide FFI onto the somata and dendrites of downstream pyramidal neurons, curbing compartment-specific activity. Although soma-targeting CCK INs basket cells have been more widely investigated (Mátyás et al., 2004; Somogyi et al., 2004; Glickfeld and Scanziani, 2006; Bartos and Elgueta, 2012; Del Pino et al., 2017; Whissell et al., 2019; Dudok et al., 2021a), non-basket cell CCK-expressing INs have been shown to target the dendrites of CA1 PNs (Cope et al., 2002; Pawelzik et al., 2002; Klausberger, 2009) and likely mediate dendritic inhibition (Basu et al., 2016; Bilash et al., 2023). Neurogliaform INs release GABA through volume transmission (Oláh et al., 2009; Armstrong et al., 2012), a non-specific form of neurotransmitter release that affects many downstream neurons within a particular radius, producing a slow, GABA_B_ receptor (GABA_B_R)-mediated inhibitory response (Price et al., 2005, 2008). Additionally, they can form gap junctions with a variety of interneuron types in area CA1 (Zsiros and Maccaferri, 2005; Armstrong et al., 2012). Studies have demonstrated that NDNF INs in area CA1 modulate learning and recall (Guo et al., 2021) and that SST INs in area CA1 modulate input-specific plasticity (Leão et al., 2012), as well as object- and fear-related memory encoding (Siwani et al., 2018). In area CA1, NPY+ neurons receive converging inputs from EC and CA3 (Milstein et al., 2015) and bistratified SST INs mediate CA3-driven FFI (Lovett-Barron et al., 2012), to shape dendritic activity and somatic firing in CA1 PNs. Therefore, NDNF INs and SST INs could feasibly modulate the integration of extrahippocampal and intrahippocampal (CA3) inputs within CA1 PNs dendrites. This may influence input-timing-dependent plasticity mechanisms (Basu et al., 2013). Neurogliaform cells may additionally modulate the soma-dendrite coupling or dendritic excitability in large populations of CA1 PNs, given that they release GABA through volume transmission and given that GABA_B_Rs are enriched in the distal dendrites of CA1 PNs (Degro et al., 2015).

## GABAergic inhibitory neurons modulate information flow in the cortico-hippocampal circuit

As described above, GABAergic INs allow for the precise modulation of pyramidal neuron activity (Basu et al., 2013; Francavilla et al., 2015; Guet-McCreight et al., 2020; Bilash et al., 2023). Within the cortico-hippocampal circuit, long-range excitatory projections from the entorhinal cortex recruit specific inhibitory neurons within the hippocampus to influence local computations. For example, Milstein et al. (2015) found that EC inputs recruit NPY-expressing neurogliaform neurons in area CA1 (Milstein et al., 2015). Using optogenetics to selectively activate projections from each EC subdivision, Li et al. (2017) found that medial entorhinal cortex (MEC) and lateral entorhinal cortex (LEC) inputs target local GABAergic INs in area CA1, including morphologically-defined axo-axonic cells, basket cells, and bistratified cells. This circuit organization likely enables EC subdivisions to drive inhibition onto the axon initial segment, soma, and dendrites of CA1 PNs, respectively (Li et al., 2017). Combining optogenetics and pharmacology, Bilash et al. (2023) found that glutamatergic LEC inputs drive strong amounts of FFI onto both the somatic and dendritic compartments of CA1 PNs by recruiting CCK-expressing INs, while also recruiting disinhibition by exciting local CR co-expressing VIP INs in area CA1 (Bilash et al., 2023).

## Disinhibitory GABAergic circuits

Beyond FFI and FBI, some particular INs can modulate disinhibition by directly inhibiting other INs and thereby activating the downstream PNs (Basu et al., 2013, 2016; Francavilla et al., 2015, 2018; Guet-McCreight et al., 2020; Bilash et al., 2023). In hippocampal CA1 area, this phenomenon can occur at the local microcircuit level and through long-range inhibitory projections (LRIPs). Locally, VIP INs are classically a disinhibitory IN subtype. However, disinhibitory roles of SST, CCK, and PV INs have also been described (Karson et al., 2009; Chamberland et al., 2023). Across the brain, most LRIPs described to date act in a disinhibitory manner by targeting local INs (Karson et al., 2009; Leão et al., 2012; Melzer et al., 2012; Basu et al., 2016; Pelkey et al., 2017; Artinian and Lacaille, 2018). Here, we provide an updated view of local and long-range disinhibitory GABAergic circuits.

## Local Disinhibitory GABAergic microcircuits

VIP INs are famous for their role in mediating disinhibition in the hippocampus (Acsády et al., 1996; Tyan et al., 2014; Guet-McCreight et al., 2020) and cortex (Lee et al., 2013; Pfeffer et al., 2013; Pi et al., 2013). They inhibit other interneurons, thereby relieving downstream principal cells from inhibitory forces (Chamberland and Topolnik, 2012; Kepecs and Fishell, 2014). Tyan et al. (2014), a pioneering study in hippocampal area CA1 that defined the functional connectivity of the interneuron-specific calretinin co-expressing VIP INs (CR+/VIP+ INs) using paired recordings and optogenetics. This study showed that when synchronously activated these VIP neurons target OLM INs to control their firing rate and timing (Tyan et al., 2014). In a more recent study, Bilash et al. (2023) used intersectional genetics to confirm that CR co-expressing VIP INs (CR+/VIP+ INs) are disinhibitory. These INs are mainly located in SLM and primarily target other INs including, CCK INs and SST INs in area CA1 (Bilash et al., 2023). Whereas the CCK+/VIP+ INs target CA1 PNs and modulate somatic inhibition. Curiously, this study found that both the disinhibitory CR+/VIP+ INs and the inhibitory CCK+/VIP+ INs are recruited by glutamatergic LEC inputs, raising the question of whether these opposing IN subtypes are differentially or simultaneously recruited during behavior to modulate cortico-hippocampal excitation-inhibition dynamics. *In vivo*, VIP INs are recruited during quiescent states and are weakly active during theta oscillations in locomotion (Magnin et al., 2019; Luo et al., 2020; Kullander and Topolnik, 2021). General optogenetic silencing of VIP INs highlight their role in goal-directed spatial learning (Turi et al., 2019).

SST INs can also play a disinhibitory role within the hippocampal area CA1 to regulate synaptic plasticity and activity of PNs. They make direct contact with PV, VIP, CCK, and CR INs in *lacunosum moleculare (LM)* as well as with NDNF INs in SR and in LM strata (Katona et al., 1999; Elfant et al., 2008; Leão et al., 2012; Artinian and Lacaille, 2018). Leão et al. (2012), observed that optogenetic stimulation of SST INs in OLM disinhibits SC inputs upon the PNs to increase the magnitude of LTP (Leão et al., 2012). A recent study showed using brief inhibition mediated by optogenetic activation of SST INs persistently interrupts firing of PV INs, so as to disinhibit spike generation in CA1 PNs (Chamberland et al., 2023).

Beyond their classical role in mediating FFI and FBI, CCK and PV INs show bidirectional synaptic coupling. Karson et al. (2009) demonstrated that PV INs anatomically project onto CCK INs and that CCK INs make functional synaptic connections onto PV INs. Such coupling exists at the level of axo-somatic, axo-dendritic, and axo-axonic synapses, between the perisomatic-targeting CCK and PV INs (Karson et al., 2009). This is of great relevance, considering that local disinhibitory circuit motifs can operate with sub-layer specific precision. This is because CCK INs show stronger inhibition upon superficial layer CA1 PNs (Valero et al., 2015), whereas PV INs preferentially target and inhibit the deep layer CA1 PNs (Lee A. T. et al., 2014). The immediate early gene, NPAS4 strengthens CB1R-expressing CCK synapses upon superficial PNs in CA1 in an experience-dependent manner (Hartzell et al., 2018). Hence CCK and PV neurons may orchestrate a fine-tuned modulation of sub-layer specific inhibition and disinhibition between the deep and superficial layer PNs in a behavioral or experience state-dependent manner. More recently, Dudok et al. (2021a) demonstrated that in area CA1, CCK and PV INs are capable of scaling their activities with respect to ensemble neuronal activity in opposite paths but both within brain states and their transitions, leading to a mechanism that PV inhibits the CCK IN activity (Dudok et al., 2021a).

In general, novel tools and transgenic rodents have allowed for the functional and circuit interrogation of the different subtypes of GABAergic neurons that play an important but non-canonical role in disinhibiting the activity of PNs. The favorable impact of rapid and context-selective disinhibitory gating of sensory and mnemonic information flow warrants further investigation into how these disinhibitory microcircuits in hippocampal area CA1 contribute to memory function and adaptive learnt behaviors relying on one trial learning and efficient recall.

## Long-range GABAergic disinhibitory circuits

While GABAergic inhibitory neurons are typically attributed to maintaining local excitation-inhibition balance and dynamically modulate activity at the microcircuit level, there is surmounting evidence that GABAergic neurons also send long-range inhibitory projections (LRIPs) between different brain areas (Jinno et al., 2007; Melzer et al., 2012; Caputi et al., 2013; Lee A. T. et al., 2014; Basu et al., 2016; Joshi et al., 2017; Melzer and Monyer, 2020; Sans-Dublanc et al., 2020; Szabo et al., 2022; Yen et al., 2022; Schroeder et al., 2023). It has been postulated that LRIPs exist to coordinate activity across longer distances within the brain and are important for generating inter-regional synchrony. An emerging principle for connectivity of LRIPs is that they usually target local GABAergic INs, thereby acting as a disinhibitory system of the principal neurons, found in the downstream target brain region (Melzer et al., 2012; Caputi et al., 2013; Basu et al., 2016; Joshi et al., 2017; Melzer and Monyer, 2020; Malik et al., 2022). Within the cortico-hippocampal circuit, LRIPs from medial and lateral entorhinal cortex (MEC and LEC), medial septum (MS), prefrontal cortex (PFC) and retrosplenial cortex (RS) have been found. In fact, LRIPs mediate bidirectionally connectivity between MEC (Ino et al., 1990; Melzer et al., 2012), MS, PFC and RS (Gulyás et al., 2003; Fanselow and Dong, 2010; Joshi et al., 2017; Yuan et al., 2017) and the hippocampus. Although LRIPs have been found to project from LEC into CA1 (Basu et al., 2016), it has yet to be determined whether similar to glutamatergic cortico-hippocampal loop there exists a GABAergic reciprocal loop counterpart from CA1 to LEC.

Hippocampal area CA1 also sends LRIPs to other sub-cortical regions as amygdala, nucleus accumbens, subiculum and band of Broca (Cohen and Squire, 1980; Jinno et al., 2007; Eichenbaum et al., 2012; Melzer et al., 2012; Caputi et al., 2013; Lee S. H. et al., 2014; Lübkemann et al., 2015; Basu et al., 2016; Witter et al., 2017; Wick et al., 2019). Given the extensive regions that interact with CA1 by sending and receiving GABAergic projections, here we discuss recent findings about the heterogeneity and functionality in circuit computations and episodic memory functions of the different LRIPs.

### Entorhinal cortex-hippocampal long-range inhibitory inputs

Within the cortico-hippocampal circuit, most LRIP axons from EC terminate in SR and SLM of hippocampal area CA1, where information is integrated in the distal dendrites of CA1 PNs (Melzer et al., 2012; Basu et al., 2016; Xu et al., 2020). Melzer et al. (2012) reported that MEC sends LRIPs to the dorsal and intermediate hippocampal areas into SLM and deep SR. These MEC LRIPs consist of axons from PV INs but likely include other GABAergic subtypes as well (Melzer et al., 2012). Although retrograde labelling experiments previously uncovered the existence of GABAergic LRIPs from EC into the hippocampus (Germroth et al., 1989), Melzer et al. (2012) was the first to address the molecular composition and functional connectivity of MEC LRIPs, as well as their potential function in modulating local GABAergic neuron theta synchrony in the hippocampus.

**Figure 4:**
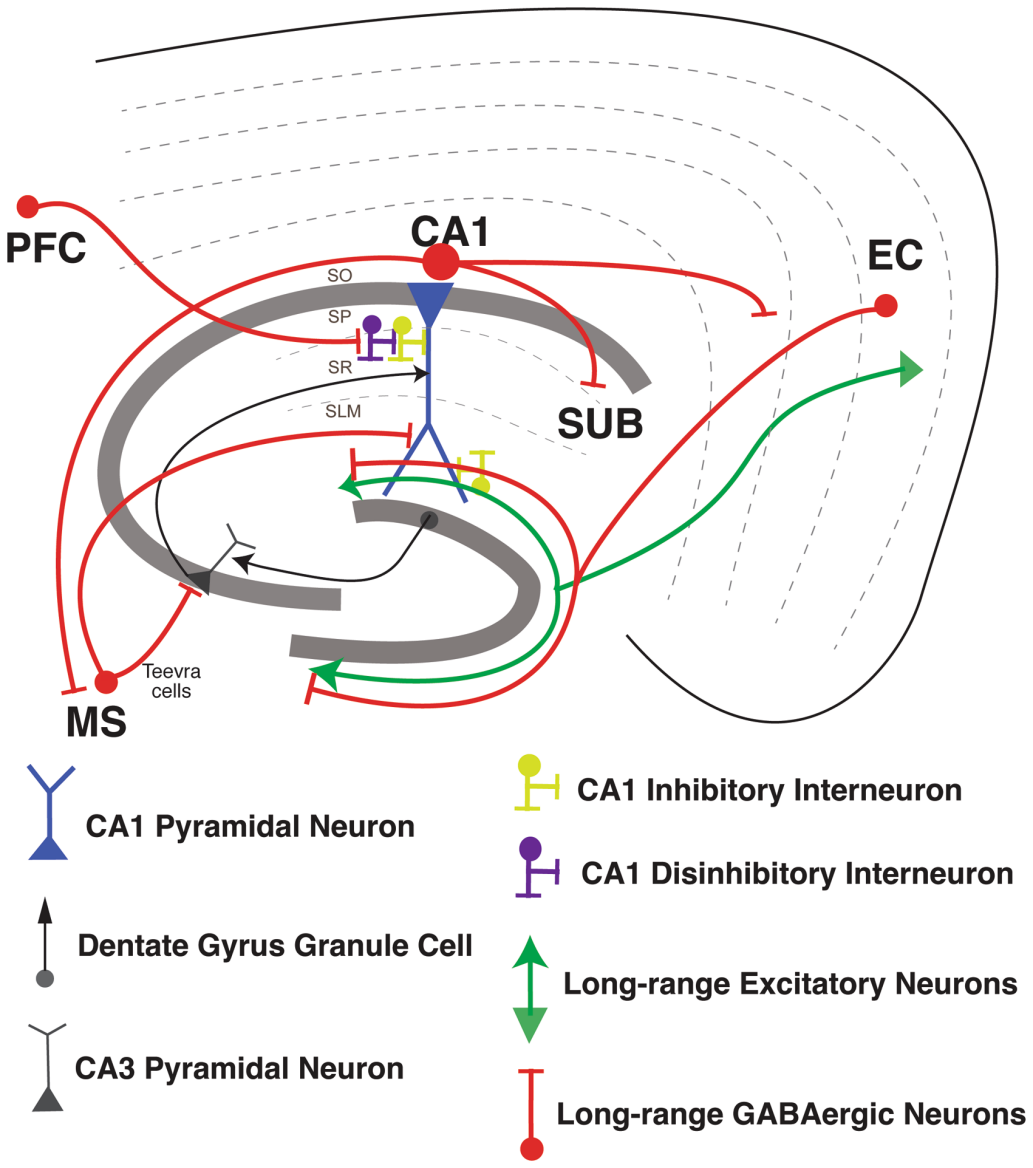
Long-range Inhibitory projections circuits in the hippocampus. Long-range Inhibitory projections (LRIPs) into hippocampal area CA1 and LRIPs outputs from hippocampal area. Inhibition or disinhibition of hippocampal CA1 pyramidal neuron (in blue), with GABAergic inhibitory interneurons (in yellow) and disinhibitory interneurons (in purple). Inputs into hippocampal area CA1 from dentate gyrus granule cells and CA3 pyramidal neurons (in black).

Basu et al. (2016) discovered that LEC also sends long-range inhibitory inputs into hippocampal area CA1. Interestingly, *in vivo* two-photon imaging of a pan-GABAergic neuron Cre line (Gad2-Cre) shows that these LEC LRIPs are strongly activated by behaviorally-salient cues such as water rewards and air-puffs. Neutral sensory cue-driven presynaptic activity in these LEC LRIP boutons is boosted supra-linearly when combined with appetitive or aversive stimuli. Accordingly, LEC LRIPs are important for novelty and contextual salience discrimination: silencing the LEC LRIPs leads to impaired novel object recognition and to the over-generalization of context-dependent fear memory. At the circuit level, LEC LRIPs prominently target Schaffer collateral-associated, dendrite-targeting CCK INs in CA1 to effectively relieve dendritic FFI. This long-range disinhibitory circuit motif facilitates the integration of EC and CA3 inputs within CA1 PNs to boost dendritic spike probability and induce input-timing-dependent plasticity. Interestingly, LRIPs from MEC and LEC are differentially distributed within hippocampal area CA1. MEC LRIPs target mostly proximal area CA1, similar to excitatory MEC projections. Meanwhile, LEC LRIPs distribute uniformly along the proximal-distal axis of CA1, in contrast to the excitatory LEC long range excitatory projections (LREP) that target mostly distal area CA1 (Melzer et al., 2012; Basu et al., 2016). It is therefore possible that long-range GABAergic projections from LEC could serve to modulate the integration of MEC, LEC, and CA3 inputs within CA1 PNs, thereby influencing the output of area CA1. This circuit organization could provide LEC LRIPs with the means to influence contextual and spatial information processing. For example, perhaps the long-range disinhibition of dendritic spikes by this salience detection circuit may contribute to context-dependent spatial tuning or remapping of place cells (Bittner et al., 2015, 2017; Figure 4).

### Other hippocampal long-range inhibitory inputs

Besides the LRIPs from the entorhinal cortex into the hippocampus, LRIPs have also been found from other brain regions that modulate hippocampal information processing (Joshi et al., 2017; Malik et al., 2022). Joshi et al. (2017) reported a new set of LRIPs neurons, named “Teevra cells,” that originate in the medial septum with general IN targets in CA3 with a few inputs into CA1. Teevra cells target axo-axonic GABAergic neurons (likely PV INs) and CCK INs in hippocampal area CA3. Teevra cells were themselves positive for parvalbumin, as well as for the transcription factor SATB1. These cells increase their rhythmicity during run and rest periods, coincident with heightened excitation in area CA1 (Joshi et al., 2017). Medial septum LRIPs are recruited for recall of contextual fear memory. Photostimulation of these projections, selectively inhibited local PV INs in area CA1, whereas chemogenetic silencing blocked memory retrieval (Sans-Dublanc et al., 2020). Furthermore, Malik et al. (2022) described a new form of LRIPs from PFC to PNs from dorsal CA1. These projections directly synapse onto VIP INs, so activating the PFC LRIPs increases FFI, enhancing the signal-to-noise ratio for hippocampus to encode object locations with increasing spatial information. Furthermore, silencing of these projections suppresses object exploration (Malik et al., 2022; Figure 4).

### Hippocampal long-range inhibitory outputs

Once information is integrated in area CA1, hippocampal output is sent back to EC, as well as to various other cortical and sub-cortical areas (Cohen and Squire, 1980; Eichenbaum et al., 2012; Melzer et al., 2012; Basu et al., 2016; Witter et al., 2017). LRIPs from the hippocampus to retrosplenial cortex (Jinno et al., 2007), amygdala (Lübkemann et al., 2015), frontal cortex (Wick et al., 2019) and nucleus accumbens (Lee S. H. et al., 2014) have also been reported. GABAergic LRIPs from CA1 have diverse molecular identities and firing properties (Melzer and Monyer, 2020). GABAergic INs originating in SO of area CA1 project to subiculum and medial septum, and increase the firing during sharp wave ripples (SWR; Jinno et al., 2007; Caputi et al., 2013; Melzer and Monyer, 2020). GABAergic INs originating in SP of area CA1 project to band of Broca and the frontal cortex, express the nNOS and NPY markers and connect to PNs and other local IN subtypes (Wick et al., 2019). GABAergic LRIPs from CA1 also project to retrosplenial cortex (RS), and to MEC, which are part of the brain’s navigation system. Both of these projections include a sub-population expressing somatostatin (Jinno et al., 2007; Miyashita and Rockland, 2007; Melzer et al., 2012). Within the CA1-subiculum circuit, LRIP from VIP INs mostly restricted in stratum oriens (SO), stratum pyramidale (SP) and radiatum (SR) of CA1 target INs in subiculum through gap junctions. These VIP IN LRIP have sparse spiking *in vitro* but are highly active during quiet wakefulness (Francavilla et al., 2018; Figure 4).

In summary, the cortico-hippocampal network for memory and navigation has bidirectional functional interactions mediated by glutamatergic and GABAergic circuits that can drive excitation, inhibition, and disinhibition in hippocampal area, locally and across long-distances. The diversity of activity-dependent and neuromodulatory tuning of specific subsets of INs contributes to a wide dynamic range for spatio-temporal modulation of balance of excitation-inhibition-disinhibition. This will allow for gating of cortico-hippocampal information flow and CA1 ensemble output by GABAergic circuits *in vivo* in a behavioral state- or task-selective manner.

## GABAergic circuits in Alzheimer’s disease

Clinical AD is staged across normal, preclinical, mild cognitive impairment (MCI), and dementia stages that are defined by worsening biomarkers, cognition, and function (Jack et al., 2018). These clinical stages are grossly associated with the spatiotemporal progression of AD pathology through the brain. In particular, memory impairment becomes first evident at the pre-dementia MCI stage when AD pathology progresses from EC to CA1, the region of hippocampus first and most affected during the course of AD (Braak and Braak, 1991; Bennett et al., 2005; Lace et al., 2009). Histological analysis has demonstrated that this correlates with synaptic loss and neurodegeneration initiated by this AD pathology in area CA1 (Price et al., 2001; Scheff et al., 2007; Kerchner et al., 2010). Molecular pathways associated with these changes relate to spine integrity, glutamate receptor loss, cellular stress response, inflammation and calcium dyshomeostasis (imbalance of the homeostasis system; Colangelo et al., 2002; Ginsberg et al., 2010, 2012; Counts et al., 2014; Hondius et al., 2016). The advent of transgenic and, more recently, knock-in rodent models of AD has further established a link between various forms of memory deficits and aspects of AD pathology within the hippocampus (Webster et al., 2014). Studies using these models have elucidated neuronal mechanisms that relate to synaptic dysfunction (Pozueta et al., 2013), aberrant synaptic plasticity (Cuestas Torres and Cardenas, 2020), and altered information coding properties (Zhao et al., 2014; Mably et al., 2017; Jun et al., 2020). Human and rodent models studies have also suggested disruption of network activity and oscillations (Zott et al., 2018; Giustiniani et al., 2022).

Excitatory pyramidal neurons have primarily drawn the focus of such cellular-level AD research, as they carry the main output from brain areas and are the principal cell type that develop neurofibrillary tangles (NFTs; Braak and Braak, 1991). Moreover, surmounting evidence of pyramidal neuron heterogeneity has suggested further studies to elucidate how these subgroups are differentially impacted by AD (Masurkar, 2018). However, as delineated above, inhibitory GABAergic neurons play a critical role in shaping and transforming information processing by pyramidal neurons through local and long-range interactions. Pyramidal neuron functional heterogeneity can in part be mediated by differential associations with specific GABAergic neuron subtypes (Lee S. H. et al., 2014; Valero et al., 2015). Therefore, the loss or degeneration of GABAergic neurons due to AD pathology can play a significant role in driving symptomatology. Moreover, reduction of GABAergic tone can increase net excitability of a network. This may impact the development and progression of AD pathology itself, as tau and amyloid can be released by activity and/or exert their effects across synapses (Kamenetz et al., 2003; Cirrito et al., 2005; De Calignon et al., 2012; Liu et al., 2012; Khan et al., 2014; Wu et al., 2016).

Broad interrogation of the GABAergic system in AD has indeed revealed relevant changes that leads to alterations. The temporal lobe in human AD features a reduction of GABA_A_ mRNA and protein, and reduced physiologic function of GABA_A_ receptors (Limon et al., 2012). Rodent AD models also show alteration of GABA function in the hippocampus (Palop et al., 2007) that can associate with disruptions in hippocampal theta oscillations and sharp wave ripples (Mahar et al., 2016; Caccavano et al., 2020), as well as disruption of hippocampal excitation-inhibition balance that impairs spatial learning (Yang et al., 2018). Moreover, recent studies in the APP-KI (amyloid-beta precursor protein-knock in) mice show that GABAergic neurons in area CA1 contribute to 30% of amyloid plaque load, highlighting the bidirectional relationship between GABAergic interneurons (INs) and AD pathology (Rice et al., 2020). While global measures of GABA_A_R dysfunction and altered excitatory/inhibitory balance were not as prominent in an analysis of human AD hippocampal regions (Scaduto et al., 2023), this does not rule out alterations in specific hippocampal IN populations and/or compensatory changes resulting from such IN-specific changes. Given the heterogeneity of IN function delineated above, here we discuss the cell type specific IN vulnerability in the hippocampus in human patients of AD, as well as in AD mouse models. A summary of these findings are described in Table 1.

**Table 1:** Summary of interneuron changes and impact in human AD and AD rodent models.

Interneuron subtype	Loss in human AD	Loss in rodent AD models	Excitability change	Network hyperexcitability	Disrupted oscillations	LTP deficits
PV	Loss in CA1	Loss in CA1	Hypoactivity	Yes	Yes	Yes
CCK	?	Inconsistent	Hyperactivity	?	?	?
SST	Unchanged in hippocampus	Loss in CA1-3	Hyperactivity	?	Yes	?
VIP CR^+^	Loss in DG	Inconsistent	Hypoactivity	Yes	?	?
NPY	Loss in DG, CA1, S	Loss in DG, CA1-3, S	?	?	?	?

Table of the heterogeneity of IN vulnerability in the hippocampus in human patients of AD, as well as in AD mouse models.

### PV INs

PV INs are the most extensively studied of the hippocampal INs in the setting of AD. While overall there is no difference in PV INs counts in AD versus control human cases (Waller et al., 2020), there is region-specific loss of PV INs and/or PV immunoreactivity limited to DG, CA1, and CA2 (Brady and Mufson, 1997; Takahashi et al., 2010). In rodent AD models, PV INs can develop tau pathology (Soler et al., 2017; Siddhartha et al., 2018), and are found to degenerate, with loss in CA1 observed most consistently (Takahashi et al., 2010; Loreth et al., 2012; Silva Albequerque et al., 2015; Verdaguer et al., 2015; Huh et al., 2016; Mahar et al., 2016; Cattaud et al., 2018; Zallo et al., 2018; Giesers and Wirths, 2020; Seo et al., 2021; Li et al., 2022). In contrast, a few studies using different amyloid and tau models show resilience (Verdaguer et al., 2015; Ahnaou et al., 2017; Sos et al., 2020; Morrone et al., 2022). Comparing the CA1 layers, PV INs in *stratum pyramidale* are most vulnerable to degeneration, followed by those in *stratum oriens* (Villette et al., 2012; Silva Albequerque et al., 2015; Mahar et al., 2016).

The degenerating or remaining PV INs appear to also have a critical role in modulating excitation-inhibition balance, plasticity, and network oscillations during the course of disease. Loss of excitatory drive to PV INs, in particular from EC Layer 2, leads to excitation-inhibition balance disruption, which can be rescued with optogenetically-driven plasticity (Xiao et al., 2017; Yang et al., 2018). Intrinsically, PV INs show an NaV1.1 channelopathy in the J20 amyloid model, which reduces their function and leads to epileptiform activity in the brain (Verret et al., 2012). With regard to plasticity, PV INs also mediate amyloid-induced suppression of CA3 → CA1 long term potentiation (Huh et al., 2016; Zhang et al., 2017). Oscillatory behavior of the hippocampus is altered in AD models, and amyloid impact on PV interneurons appears to play a critical role (Hollnagel et al., 2019; Chung et al., 2020; Park et al., 2020).

PV INs also appear to have relationship to AD pathology that are intriguing areas for future work. Through their impact on network excitability, PV INs not only promote hyperexcitability, a process that may give rise more pathology, but also can induce vulnerability to further amyloid-induced damage (Hijazi et al., 2019, 2020). Moreover, in APP/PS1 mice (AD model with human amyloid-beta precursor protein and presenilin-1 mutations), PV INs show close interactions with microglia (Gervais et al., 2022). However, another study has called in to question the relationship between PV INs and AD pathogenesis (Mackenzie-Gray Scott et al., 2022). Of note, PV INs also express amyloid-degrading enzymes which would indeed be counterintuitive (Pacheco-Quinto et al., 2016).

While PV INs have been extensively studied in the context of AD, many further questions have arisen. In particular, reconciling how they promote hyperexcitability while also suppressing plasticity is needed to better understand how chronic PV IN dysfunction alters the course of disease. Furthermore, PV INs are themselves heterogeneous, and it is not clear if subtypes are differentially affected. Notably, in 5 × FAD mice, an AD model harboring 5 familial AD mutations, the soma-targeting PV basket cells are affected, reducing their activity during sharp wave ripples, whereas the bistratified and axo-axonic subpopulations were unchanged (Caccavano et al., 2020). Lastly, there has been limited study on the physiologic impact of tauopathy on PV INs.

### CCK INs

CCK hormone, highly expressed in the hippocampus, has been suggested to enhance healthy mnemonic functions and serves as a biomarker of cognitive and neuronal integrity in neurodegenerative disease (Plagman et al., 2019; Zhang et al., 2022). There have been few studies specifically examining CCK INs, relative to their extensive study in non-pathologic settings, and these are primarily limited to rodent models featuring or exposed to amyloid pathology. Those examining CCK expression or total number show varied results. Whereas intraventricular injection of amyloid does not alter CCK mRNA levels in the hippocampus (Aguado-Llera et al., 2018), and the number of CCK INs in CA1 (in SO) remained unchanged with intrahippocampal injection of amyloid (Villette et al., 2012), APP/PS1 mice feature a reduction of CB1R-expressing CCK interneurons in DG and CA1 (He et al., 2021). While these differences may arise from exogenous versus endogenous exposure to amyloid, it is also possible that CCK IN subpopulations show a differential vulnerability. In addition, CCK INs appear to undergo physiologic changes to their synaptic drive and overall excitability. In the APP KI model, these interneurons feature signs of reduced inhibitory input (Petrache et al., 2020; Shi et al., 2020) and show early hyperactivity that precedes amyloid accumulation, which is then followed by reduction in GABA production (Shi et al., 2020). The above studies suggest that AD-induced changes in CCK INs function may play a critical role in cognitive deficits and promoting disease progression. Of note, cannabinoids (CB) that target CB1R-expressing CCK INs show promising effects in reducing amyloid plaque deposition and hyperphosphorylation of tau (Aso and Ferrer, 2014; Aguirre-Rueda et al., 2015; Abate et al., 2021; Xiong and Lim, 2021; Khavandi et al., 2023). As such, further studies examining subtypes of CCK INs and their impact on function and pathology are needed. Moreover, the relationship between tau pathology and these interneurons have not been studied, despite suggestion of a relationship in human AD (Lenders et al., 1989). Further study of CCK INs in human AD is also needed.

### SST INs

As a critical modulator of pyramidal neuron dendritic excitability, SST INs have been the focus of several studies. Importantly, there is no difference in SST INs counts in AD and control human cases (Waller et al., 2020), suggesting that they may be largely resilient to neurodegeneration. This finding is recapitulated in the TauPS2APP (Loreth et al., 2012) and hAPP (Palop et al., 2007) rodent models. In contrast, loss of SST mRNA is found with intraventricular injection of amyloid in rat (Aguado-Llera et al., 2018), and SST INs loss appears in the mouse 5xFAD and PS1xAPP and rat TgF344-AD models (Ramos et al., 2006; Li et al., 2022; Morrone et al., 2022). A reason for these discrepancies, beyond choice of animal model, may because SST INs vulnerability is location- and subtype-specific. Indeed, hippocampal amyloid injection causes loss of subset of SST INs in CA1 *stratum oriens* that do no co-express NPY or PV (Villette et al., 2012). In the TgCRND8 amyloid model, there is a subregion- and lamina-specific decrease of SST-expressing interneurons primarily in *stratum oriens* of CA1, CA2 and stratum radiatum of CA3 (Silva Albequerque et al., 2015).

SST INs may also play a more active role in AD as other studies have evaluated SST INs physiology and relationship to AD pathogenesis. For example, CA1 SST INs show early hyperactivity in the APP KI model preceding amyloid accumulation, which is then followed by reduction in GABA production (Shi et al., 2020). Relatedly, the impact of A-beta oligomers on hippocampal oscillations is suggested to be mediated by effects on SST INs (Chung et al., 2020; Park et al., 2020). In APP/PS1 mice, SST INs show increased interactions with microglia (Gervais et al., 2022). Interestingly, SST INs may directly modulate AD pathology. SST INs express amyloid-degrading enzymes (Pacheco-Quinto et al., 2016). Examination of human AD pathology suggests a relationship of SST INs to the development of tau pathology (Lenders et al., 1989).

In summary, SST INs present an intriguing target for modulating AD pathophysiology due to their targeting of pyramidal neuron dendrites, partial resilience to neurodegeneration, and potential influence over amyloidosis and tauopathy. Open questions remain over their exact influence on cognitive and behavioral deficits in the context of hippocampal circuits and the efficacy of leveraging this knowledge to improve hippocampal function and counteract AD pathogenesis.

### CR co-expressing VIP INs

There are several studies examining CR-expressing INs, which often co-express VIP and mediate disinhibition, as discussed above. In human AD hippocampus, loss of CR immunoreactivity is found in DG (Takahashi et al., 2010). In rodent models of AD featuring amyloid or both amyloid and tau, evidence is inconsistent that CR INs degenerate in various hippocampal subregions (Baglietto-Vargas et al., 2010; Takahashi et al., 2010; Loreth et al., 2012; Aguado-Llera et al., 2018; Zallo et al., 2018; Giesers and Wirths, 2020; Shi et al., 2020; Li et al., 2022). Physiologic characterization has demonstrated that in CA1 they have unchanged intrinsic excitability but show hyper-inhibition through upregulated purinoreceptors in an APP KI model (Shi et al., 2020). Given their targeting of interneuron, this associates with pyramidal neuron hyperexcitability. Together, these results imply that CR-expressing INs may be spared during AD in some subregions, and could thus be a potential target for pharmacological and therapeutic intervention. Furthermore, there is promising evidence that VIP peptide secreted by VIP INs alongside GABA contributes to neuroprotection and neurotrophic processes, and inhibits Aβ induced neurodegeneration by suppressing microglial secreted neurotoxic factors (Song et al., 2012; Korkmaz et al., 2019). Further studies are needed to more directly elucidate the impact of altered disinhibition by CR, VIP, and CR^+^/VIP^+^ INs populations on hippocampal function and related behaviors.

### NPY INs

As mentioned above, NPY expression defines a heterogeneous population of INs that co-express other molecular markers and have unique functions in non-pathologic settings. Most work on NPY interneurons has been centered on quantifying expression levels or neurodegeneration. In human AD there is a reduction in NPY that is most prominent in the DG hilus, CA1, and parasubiculum (Chan-Palay et al., 1986). Intraventricular injection of amyloid in rat reduces NPY mRNA levels in the hippocampus (Aguado-Llera et al., 2018). At a subregion level, the human pattern is largely mimicked in rodent AD models with amyloidosis and dual pathology, with some models also demonstrating CA3 and CA2 vulnerability (Palop et al., 2007; Loreth et al., 2012; Silva Albequerque et al., 2015; Mahar et al., 2016). Additional complexity to alteration of the NPY INs network is suggested by laminar-specific vulnerability, with stratum pyramidale most consistently vulnerable (Villette et al., 2012; Mahar et al., 2016), alterations of NPY-subtype expression (Palop et al., 2007), and findings that NPY-expression identifies SST INs most vulnerable to degeneration (Ramos et al., 2006). Open questions remain on the impact of this region-specific vulnerability on hippocampal function and related behaviors. Furthermore, as NPY can subcategorize other IN types, it is unclear if AD-related physiologic alterations also occur based on NPY expression. Additional work is also needed to investigate possible changes in their excitability and impact of this on plasticity and network activity.

## Conclusion

**Figure 5:**
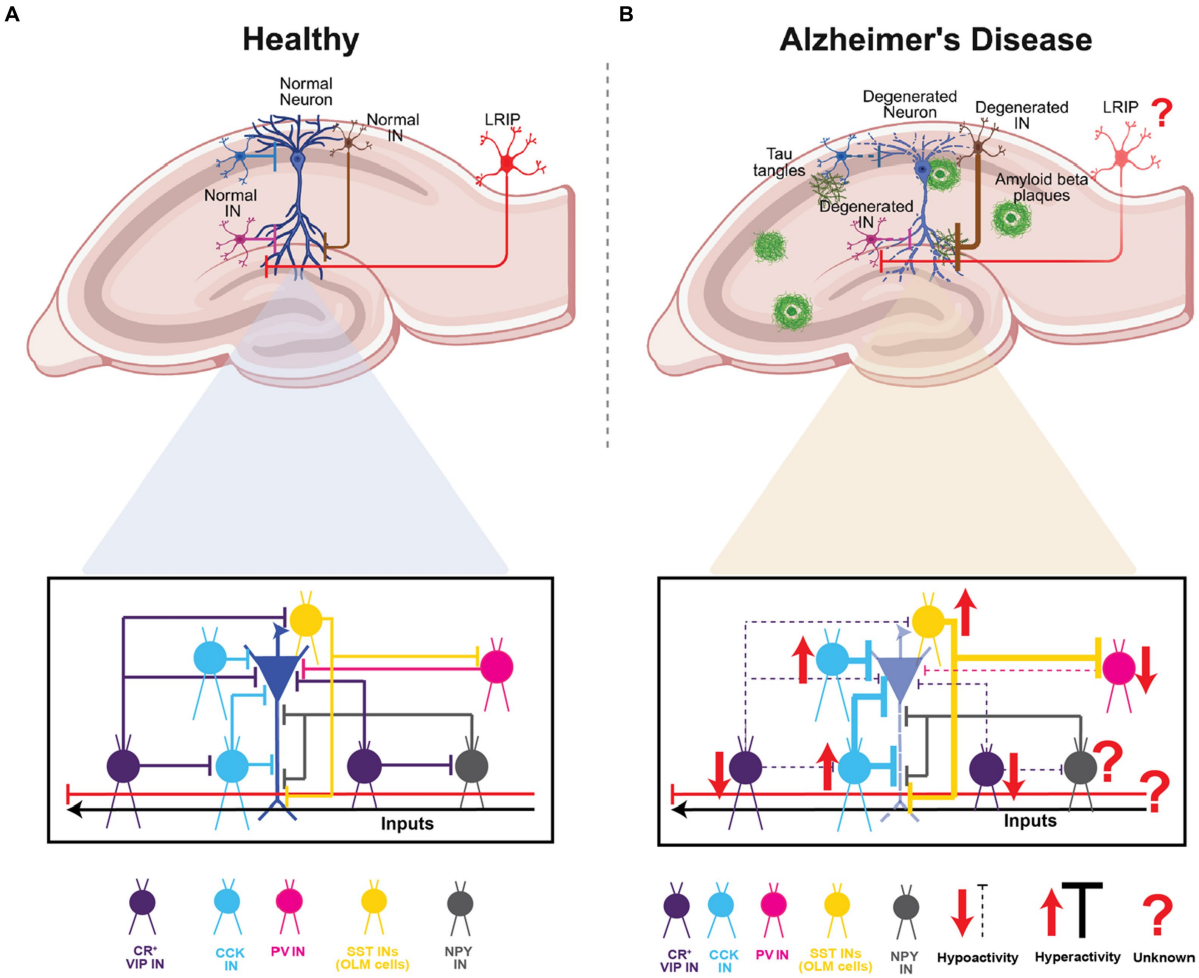
Summary of CA1 INs in health and Alzheimer’s disease. **(A)** Schematic representation of the hippocampal area and a normal pyramidal neuron of CA1 highlighting the INs subtypes and the inhibition and disinhibition processes in healthy circuit. **(B)** Schematic representation of the hippocampal area and a degenerated pyramidal neuron of CA with amyloid plaques depositions and Tau tangles. In lower panel highlighting the INs subtypes and the dysregulation of hyperexcitability or hypoexcitability processes found in AD. Top panels of **(A,B)**, created with BioRender.

Given their diversity, GABAergic circuits play a wide range of critical roles – from modulation of single-neuron and network activity in a compartment- and layer-specific manner, to acting as gain control switches or knobs to modulate the dynamics between excitation-inhibition-disinhibition, to synchronizing distant brain areas and creating spatio-temporal windows for integration, coincidence-detection and signal-to-noise modulation. As highlighted above, there is surmounting evidence that specific GABAergic circuits in the hippocampus may be involved in the pathology and progression of AD. In this context, several open questions remain regarding their differential engagement with pyramidal neuron subtypes (Masurkar, 2018), their role in dysregulated information processing, plasticity, and network activity, and their impact on behavioral deficits and disease progression. Notably, while their specific role in AD is yet to be explored, the more recently discovered long-range GABAergic projections support inter-regional coordination of activity and context discrimination. Given their disinhibitory role in area CA1, the dysfunction of these LRIPs in AD might disrupt spatial and non-spatial memory processing. These modifications, may elicit hyperactivity and increased plasticity to exacerbate the spread of AD pathology and neurodegeneration. This warrants future studies using mouse models of AD as well as proteomics and transcriptomics-based studies in human patients to examine the pathological vulnerability of specific types of long-range inhibitory projections (regional: MEC vs. LEC; cell-type: SST- vs. PV- vs. VIP-expressing) and their downstream interactions. Such information will be important for correlating their dysfunction with disease pathology and cognitive and physiological phenotypes and identifying potential targets for therapeutic intervention (Figure 5).

## Author contributions

MH-F, AM, and JB: planning and conceptualization. MH-F, OB, AM, and JB: manuscript preparation. MH-F prepared the figures with input from OB and JB. AM prepared the table. All authors contributed to the article and approved the submitted version.
